# Miscellany

**Published:** 1911-11

**Authors:** 


### MISCELLANY

### Progress of the United States

POPULATION 1800, 5 1-3 million; 1910, 93 3-4 million. Area 1800, 843,255 square miles; 1810, 1,734,630; 1850, 2,995,536; 1853, 3,026,789, since which date there has been no increase in the continuous area of the continental states.

Per capita debt, 1800, $15.63; 1865, $76.98; 1911, $10.83. Per capita circulation of money, 1800, $4.99; 1911, $34.35 (but probably having the purchasing power of only about $10 in 1800).

Excerpts from Statistical Record of the Progress of the United States, 1800—1911, which can be had by application to the Bureau of Statistics, Department of Commerce and Labor, Washington, D. C.

AUTOMOBILE EXPORTS.—In 1901, less than a million dollars' worth of automobiles were exported from the United States. In 1910, the amount was thirteen million. Calculated by increase for the first seven months of 1911, and including automobile parts and tires, the total will reach twenty million for 1911. Meantime, the average price, per machine has fallen from $2,000 for 1908 to about $1,200 for 1911. About 40 per cent. of the exports go to Canada.

INTERNATIONAL LIST OF CAUSES OF DEATH.—Until 1893, no two countries used the same forms and methods for compiling mortality reports. In 1907, Bertillon estimated that the uniform International system applied to over two hundred and twelve million of population. Since then, the registration area as well as the population of the United States have increased and Great Britain has been added. The entire western hemisphere, practically all British possessions and dependencies, China, Japan and many European countries now employ the uniform system. All physicians are urged by the Census Bureau, to study the International List of Causes of Death and to coöperate toward securing reliable statistics by exercising the utmost thoroughness in filling in death certificates, in accordance with instructions issued by local boards of health in coöperation with the general authorities.

---

THE NEW YORK STATE DEPARTMENT OF LABOR, Bureau of Labor Statistics, calls attention to Chapter 258 of the Laws of 1911, which requires medical practitioners in this state to give immediate notice to the Commissoner of Labor at Albany of every case attended by them of *poisoning by lead, phosphorus, arsenic or mercury* and every case of *anthrax* or of *compressed air illness* (the latter commonly known as the "bends") if such disease was contracted as a result of the nature of the patient's employment. Failure to give such notice renders a practitioner liable to a fine.

The notice must state the name of the patient, his postal address, his place of employment, his occupation, trade and the disease from which the patient is suffering.

Blank forms will be supplied by the Bureau on application.

---

BUSINESS WOMEN.—Dr. Eugenia Hancock of New York, endorses the advice of Dr. Martha Lovell of Boston, against women entering into business life. This reminds us of certain remarks which our friends have made regarding discrepancies between practice and theory in diet. Dr. Hancock describes the ocular, nervous and general physical break-down of the business woman between 40 and 50. Far be it from us to advocate a general abandonment of household duties and domestic life on the part of women. Still we can call to mind a good many business and professional women who are active, healthy, happy and young looking at the age mentioned, and a good many housewives who are prematurely old, physically and mentally in a state of decadence, at the same age.

---

A CONVENIENT FORM FOR KEEPING BISMUTH-PASTE READY FOR USE.—The following method of putting up bismuth-paste is convenient, and meets all the rigid requirements of strict asepsis:—Small collapsible tubes, such as vaseline is dispensed in, are procured. These tubes differ from vaseline tubes in that they end in a pointed snout, over which a protective cap screws. The tubes are sterilized by dry heat, and are filled with the paste, after it has been sterilized. The open end of the tube is closed by folding it on itself several times. The paste is used by removing the screw-cap, inserting the nozzle into the fistula, and forcing the paste in by finger-pressure on the tube.

M. G. Seelig, St. Louis, *Interstate Med. Jour.*

---

Harvesting and Distilling Wintergreen.—Some notes of a trip through Monroe County, Pa., the principal wintergreen producing section of the State, published in the *American Perfumer,* make interesting reading.

The producing cost depends almost entirely on the cost of gathering the raw material. The leaf pickers were formerly paid about $1 for 100 pounds, but this price has risen till at the present time expert pickers cannot be obtained for less than $2 to $2.25 a hundred pounds. A good picker can gather this quantity in one day. The wintergreen plants grow to a height of three to four inches, though the stems, some seven or eight inches long, trail for half of their length.

There are about sixty stills distributed over Monroe County, in a section about ten miles by twenty miles. Some of these stills have permanent locations, while others are moved from place to place, as the leaves in the neighborhood are exhausted.

---

Denatured Alcohol.—Denatured alcohol is esentially a farm product. It may be distilled from potatoes, corn, cassava, beets, sugar-cane and other starch and sugar-bearing grain, plants and vegetables. It is, therefore, simply the ordinary alcohol of commerce, freed from the internal revenue tax, when made unfit for use as a beverage. The United States internal revenue department statistics for the fiscal government year ended June 30, 1910, show that there were used in that period 10,598,749.2 gallons as compared with 7,971,636.4 gallons for the fiscal year ending June 30, 1909.

The Department of Agriculture has just published a report advising the use of potato culls for making industrial alcohol.

The spirit yielding material in vegetable matter is its fermentable contents. It has been demonstrated that one hundred pounds of the following farm products will produce alcohol in the following quantities: Rice, six wine gallons, 190 proof; rye, barley, spelt corn and sorghum seed, 5 gallons; Irish potatoes, 1¼ gallons; sugar beets, 2 gallons; sorghum or sugar cane, 4 gallons; waste molasses, 6 gallons; grapes, 2 1-6 gallons; banana, 4 4-5 gallons, and other fruits from 1 to 1½ gallons.

JOHN G. CAPERS, Commissioner.

### Educational Opportunities in Chicago

UNDER the above title, the Council for Library and Museum Extension, representing the municipal Board of Instruction, various libraries, the park commission, universities and other higher educational institutions located in Chicago, museums, certain clubs and societies having philanthropic objects, have published a pamphlet describing the manifold opportunities for self-education open to the public. Publicity and coördination are the general divisions of the work of this council: Publicity meaning rather the informing of the public as to what educational advantages are before them either free or at nominal cost, than the exploitation of the city to the country at large. Various institutions not directly represented in the council, as the Y.M.C.A., musical organizations, lecture clubs, L'Alliance Francaise, an important Catholic college, etc., are described or mentioned in the pamphlet.

Chicago is a large city, important commercially and economically, but also in intellectual and cultural matters. Though not so large as New York, it impresses a stranger as being, in some respects, greater, at least in the unity and far-sightedness of its public policy.

Education of a voluntary nature, of adults or of youths of high school and college age, who must earn a living, is an important factor in every community. Granted the ordinary foundation of compulsory education in force in most enlightened communities, and a fair degree of intelligence, there is scarcely any text book study, language, history, logic, etc., natural science or other branch not requiring technical laboratory training and constant preceptorship, which cannot be gained by access to good libraries, museums, lectures, combined with field work available to a greater or less degree for each science, according to accidents of environment. What is needed in most cities, and what, as we understand it, the Chicago Council aims especially to supply is a helping, guiding hand and a systematizing of the educational material available. Without such help and systematic coöperation, the public merely sees a lot of curiosities in museums, strange plants in green houses, a free menagerie, and is diverted by stereopticon shows.

Another highly important function of municipal educational institutions is that it keeps people out of mischief. With abundant leisure after the modern short working day, with exaggerated notions of liberty and distorted ideas of politics, idleness may not merely lead to personal dissipation and deeds of violence, but to serious organized scheming against the essentials of law and good order. While, piece meal, libraries, museums, zoos, horticultural displays, free evening entertainments of a literary and scientific nature, may do much in this direction, they cannot give the balance and sustained interest of a genuine, systematic education.

During the preliminary agitation of the establishment of a collegiate department of the University of Buffalo, we took an inventory of the educational material in libraries, museums, etc., available and reached the conclusion that, for everything except actual class room instruction, the city already had the equivalent of a university rather beyond the average. Chicago's example might well be followed—and the plan extended and intensified —by every city in the country. Even very small towns could greatly increase their civic usefulness by coöperation on the part of disjointed units of educational work.

---

DR. JOHN W. DRAPER (*Indianapolis Medical Journal*), made the first photographic likeness of any person in the country. The *Literary Digest* for May 13, says that the model for the first portrait which was taken in 1839, was Dr. Draper's sister and his substitute for the modern camera was an old cigar box with a spectacle lens. The subject had the pretty name of Dorothy Catherine Draper. Jacques Daguerre invented the process that bears his name. Daguerrotypes are now simply mementos.

---

THE Medical Department of the Tulane University of Louisiana, announces the inauguration of a Department of Tropical Medicine, Hygiene and Preventive Medicine, beginning October 1, 1911, in charge of Professor Creighton Wellman and Staff.

Laboratory courses, clinics and lectures will be given in the regular junior and senior classes, and, in addition, graduate courses are offered, for which certificates will be issued, counting towards special degrees to be created as soon as the Tulane School of Tropical Medicine is in force.

---

THE CLIMATIC AND HYGIENIC INFLUENCE OF FOREST GROWTH.

James M. Anders, Philadelphia, Pa., states that forests are useful as modifiers of extremes of temperature, especially the daily range, and as rendering summer less sultry and winter less severe. They are natural conservers of aqueous vapor, thus maintaining an unvarying degree of atmospheric humidity, and are natural producers of ozone, which removes the natural organic impurities from the atmosphere by oxidation.—*Medical Record,* September 30, 1911.

